# Childhood Socioeconomic Position and Cardiovascular Disease Among Older Women and Men: The Moderating Role of Parenthood Onset

**DOI:** 10.3389/ijph.2022.1604884

**Published:** 2022-11-28

**Authors:** Manuel Ortiz-Llorens, Ignacio Cabib, Claudia Bambs

**Affiliations:** ^1^ Escuela de Obstetricia, Facultad de Ciencias, Universidad Mayor, Santiago, Chile; ^2^ Instituto de Sociología & Departamento de Salud Pública, Pontificia Universidad Católica de Chile, Santiago, Chile; ^3^ Centro UC Estudios de Vejez y Envejecimiento, Pontificia Universidad Católica de Chile, Santiago, Chile; ^4^ Departamento de Salud Pública, Facultad de Medicina, Pontificia Universidad Católica de Chile, Santiago, Chile

**Keywords:** gender differences, cardiovascular disease, life course, childhood socioeconomic position, parenthood transition, old age, life history calendar, Chile

## Abstract

**Objectives:** Based on a life-course approach, the purpose of this study is to analyze how the age at the birth of a first child moderates the relationship between childhood socioeconomic position (SEP) and cardiovascular diseases (CVD) incidence in old age, separately for women and men.

**Methods:** We used a rich and representative life history survey of people aged from 65 to 75 living in Santiago, Chile (*n* = 802), and weighted multivariate statistical models. Data collection process involved the use of face-to-face life history calendars, administered by well-trained interviewers.

**Results:** Early motherhood increases the risk of suffering CVD among older women with a disadvantaged childhood SEP, while late motherhood decreases it. By contrast, early fatherhood decreases CVD risk among older men with an adverse childhood SEP, while late fatherhood increases it.

**Conclusion:** Our findings about the moderating role of parenthood onset on CVD risk among older women and men with a disadvantaged childhood SEP contributes to public health reflections on unexplored cardiovascular risk factors, which lead to substantial changes in women’s and men’s life courses, and might optimize cardiovascular prevention strategies.

## Introduction

Cardiovascular diseases (CVD) are the main cause of death worldwide [1]. Ischemic cardiomyopathy or acute myocardial infarction (AMI) and cerebrovascular accident (CVA) are the most prevalent pathologies over the last 2 decades, causing over 15 million deaths annually [2]. In Chile, these diseases group also leads causes of death at 27.0% [3].

Life course studies have provided evidence to understand how socioeconomic circumstances in early life stages, along with exposure to multiple experiences in different domains during individuals’ lives (e.g., education, family, work, health, and others) cumulatively affect CVD development in both women and men [4, 5]. Specifically, these studies have shown that although these pathologies are rare in young people, the pathophysiological process underlying CVD comes from exposure to multiple social disadvantages beginning in very early life stages [5, 6].

For instance, it has been shown that people who faced an adverse socioeconomic position (SEP) in childhood have greater CVD risks, and are almost three times more likely to die from CVD in old age, compared with people who did not face these economic difficulties.1 This has been attributed, among other factors, to the fact that early adverse socioeconomic conditions lead to lower education and subsequently worse economic conditions in adult life [7–9]; to the presence of riskier behavior impacting health over time, such as tobacco use and alcohol consumption [10]; as well as lower access to health diagnosis and treatment [11].

Becoming a mother or a father, and the timing in which this occurs, is a life experience which is highly related with childhood SEP, as well as with the later health of people [12, 13]. Poor SEP in early stages increases the chances of early parenthood [14, 15], which in turn often interrupts parents’ educational paths, thereby reducing their adult socioeconomic outcomes [16, 17]. Studies in multiple developed countries have also shown that very early parenthood (before age 20) leads to both women and men facing greater risk of CVD (including arrhythmia and AMI) as well as increased allostatic loads in old age [18, 19]. Most studies have centered on women, given the plausible biological association between early pregnancy and CVD presence due to greater risks of eclampsia, pregnancy-related hypertension, insulin resistance and altered cholesterol profiles, among other factors. However, the same studies provide indications of the relevance of also considering men in study samples, in order to contrast biological explanations with social or behavioral explanations [19, 20].

Despite massive evidence regarding the association of both disadvantaged childhood SEP and early motherhood and fatherhood with greater CVD risk in later life stages, to our knowledge there are no studies evaluating these three factors’ association. Consequently, it is unknown for both women and men what the simultaneous impact of adverse childhood socioeconomic conditions and early parenthood may be on later CVD presence. Simultaneously analyzing these factors is relevant given that the negative effect of early socioeconomic adversity on CVD could be compensated by later parenthood; or, by contrast, the negative effect of an economically disadvantaged childhood could be increased by early parenthood. Finally, the interaction between these factors could follow a different path in women and men.

Based on a life-course approach [21], the purpose of this study is to analyze how age at the birth of a first child moderates the relationship between childhood SEP and CVD incidence in old age, separately for older women and older men. Based on previous literature, we expect that the association between disadvantaged childhood SEP and CVD is intensified in older women and men who had their first child very early, and that this effect will be stronger among older women.

## Methods

### Data and Study Sample

This study used data from the representative and retrospective life history survey “Life course and vulnerability among older people in Santiago, Chile.”’ This is the first Chilean survey to gather annual retrospective information on multiple dimensions of the life course. The study sample is composed of 802 people between 65 and 75 years old residing in Santiago, the capital of Chile, which accounts for 40.5% of total national population.

Following the latest quality standards of data collection defined by the American Association for Public Opinion Research [22], and drawing on a sampling frame of individuals aged 65–75 in Santiago provided by the National Institute of Statistics, the sample of 802 individuals was randomly selected and recruited in four stages: 1) blocks within the 32 communes of Santiago, 2) households within blocks, 3) households within the dwelling (if there is more than one household within a dwelling), and 4) a person aged 65–75 within the selected household or dwelling. Assuming a simple random method to select the sample, a maximum variance (*p* = 0.5), and a 95% confidence level, the total sample error is estimated to be ±3.5 points for an infinite population.

To avoid potential selection biases, the study sample was weighted with an expansion factor correcting estimates based on known characteristics of the age range between 65 and 75 years in Santiago (such as zone of residence, education level, and gender).

### Procedure

We conducted the survey between March and August 2019. The data collection process involved the use of face-to-face life history calendars (administered by well-trained interviewers), which helped respondents remember and chronologically organize various episodes throughout their lives along with the approximate dates of occurrence.

To increase the measurement quality of the life history calendar, eight cognitive interviews and four usability tests were conducted during the questionnaire pretest phase among men and women from different educational backgrounds. Finally, following the latest AAPOR’s quality standards, the cooperation rate was 88.8%, response rate was 66.5% and rejection rate was 8.3%.

### Measures

#### Dependent Variable: Cardiovascular Disease

The dependent study variable is the presence or absence of heart diseases and/or cerebrovascular diseases. Following international standards, participants were asked “Has a doctor ever told you that you have or had any of the health problems shown on this card. Remember that they can be health problems which you currently have or previously had.” The options included “Heart attack, including myocardial infarction or coronary thrombosis, or any other heart problem, including congestive heart failure,” as well as “Stroke or vascular brain disease.”

Based on respondents’ replies about CVD background, we built a binary indicator with a value of “1” in the case of medical diagnosis of heart diseases and/or cerebrovascular diseases, and “0” in the case of not reporting any of these diseases.

According to the latest findings from the Chilean National Health Survey in 2017 [23, 24], among the population aged over 65, 10.0% have suffered a heart attack, 8.2% have suffered a stroke or vascular brain disease, 73.3% have high blood pressure, and 65.6% are at high cardiovascular risk using the Framingham score [25].

#### Independent Variable: Childhood Socioeconomic Position

Study participants were asked: “Now I would like you to recall the time when you grew up, that is, from when you were born up to age 15. Would you say that your family’s economic situation was rather good, around average or poor?”. Answer alternatives were “Rather good,” “Around average,” “Poor,” and “Varying.” With this information, a two-level categorical variable was constructed: 1) “Low SEP” (when respondents reported the “Poor” alternative) and 2) “Upper-Middle SEP” (when they selected any other option).

#### Moderator Variable: Age at Birth of First Child

The moderator variable is “age at birth of first child” (ABFC, hereinafter), which was used both as a continuous and as a categorical variable. The continuous measurement corresponded to the age (in years) at which each respondent had their first child. The categorical measurement was constructed according to ABFC distribution by gender, based on the average obtained for men and women, with the two following values: “ABFC below average” (women ≤22 years; men: ≤25 years) and “ABFC above average” (women ≥23 years; men: ≥26 years). Consequently, while continuous ABFC measures the fact of having a first child later, categorical ABFC measures the fact of having begun parenthood earlier or later compared to same-gender peers.

#### Covariates

Analyses were adjusted by the following sociodemographic variables: age (years), educational level (“primary or less” and “secondary or more”) and number of children. Analyses also included traditional cardiovascular risk factors, such as a background of medically diagnosed arterial hypertension or high cholesterol, and the number of years in which the person reported daily tobacco consumption. Yet, we also estimated our analyses not including covariates that may be in the causal midway between childhood SEP and CVD, that is, arterial hypertension, high cholesterol, and tobacco consumption (see Supplementary Table S1). As seen, when excluding these covariates, the association between childhood SEP and CVD presence remain statistically significant among the group in which we found significant findings in the main results, that is, men (see Table 2 below).

### Statistical Analysis

A weighted bivariate statistical analysis was conducted first to explore gender differences in all study variables. Then, weighted logistic regression models were conducted to evaluate the association between all variables and CVD presence, among men and women separately (results presented in odds ratios [OR] with a Confidence interval of 95%). To simplify the interpretation of our results, we calculated the average marginal effects (AME) of the interaction between childhood SEP and ABFC (both categorical and continuous). Also, besides the evidence support we found in previous literature to stratify our analyses by gender, in Supplementary Table S2 we show our regression analyses over the general sample, also including interaction effects between childhood SEP and gender. As shown, the results on the general sample indicates no statistically significant associations neither between childhood SEP and CVD, nor between ABFC (both categorical and continuous) and CVD. Interactions effects between childhood SEP and gender over CVD are not statistically significant either. These findings confirm the need to stratify our analyses by gender. All statistical analyses in this study were conducted using STATA 14.1 software.

### Ethics Statement

This research project has been approved by the Ethics Committee of the Faculty of Social Sciences at Pontificia Universidad Católica de Chile (institutional review board [IRB] approval number: 210612002), which conforms to the provisions of the Declaration of Helsinki, the Declaration of Singapore, and the Nuremberg Code. Informed consent was obtained from all subjects involved in the study.

## Results

### Sample Characteristics

**FIGURE 1:**
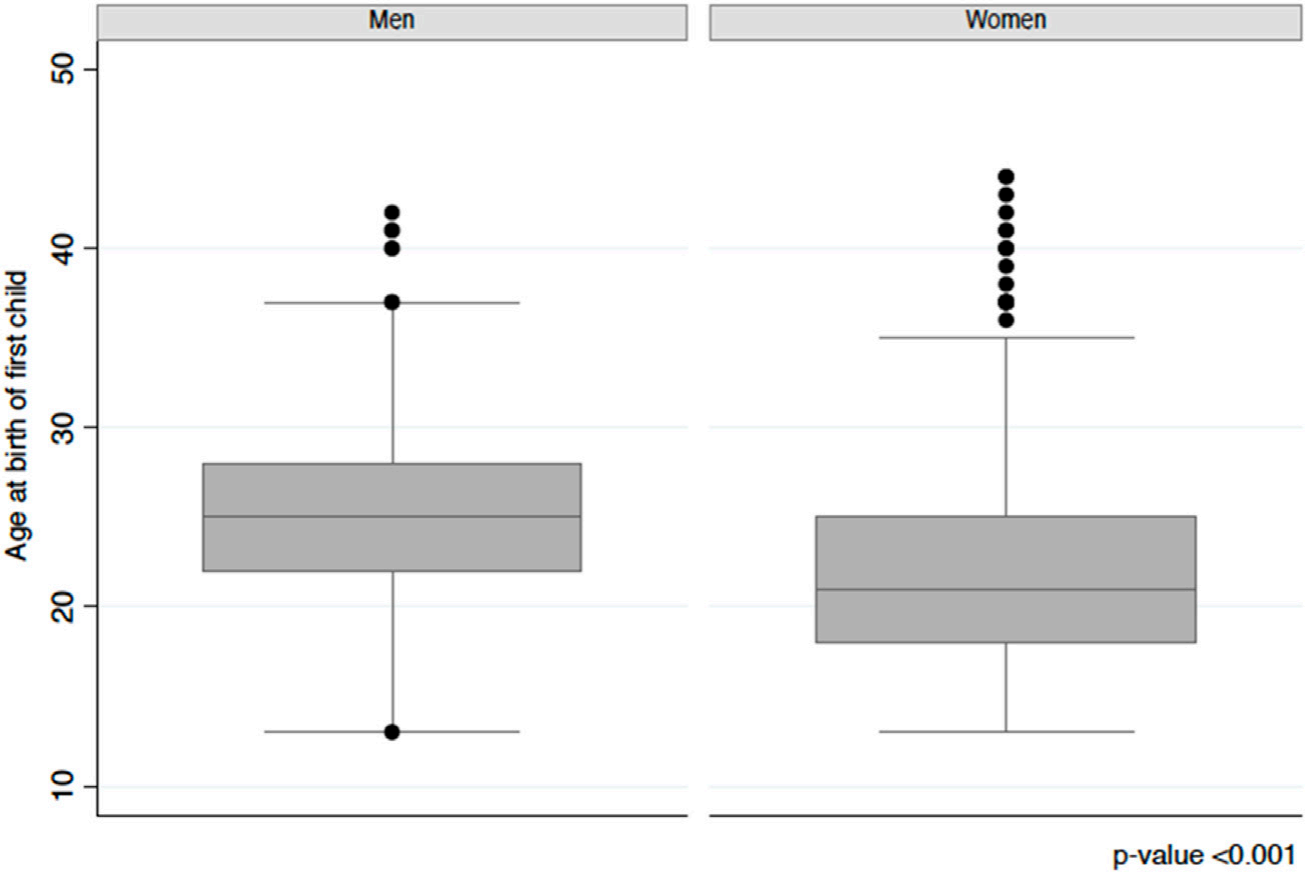
Average age at birth of a first child among men and women (Santiago, Chile. 2019).

Table 1 shows a weighted characterization of study participants by gender. As we can see CVD prevalence, considering both heart and cerebrovascular diseases, was higher in older men than in older women (27.9% and 17.7%, respectively), without significant gender differences, which is consistent with national figures [23, 24]. A relevant proportion of respondents said that they had a low SEP during the first 15 years of their lives (men: 37.1%, women 40.8%). Continuous ABFC presented highly significant gender differences (Figure 1). Women became parents, on average, 3 years before men. 55.0% of them had their first child before age 22. By contrast, men became parents later (age 25.8 years). Regarding the control variables, the proportion of older women with an education level of primary or less was significantly higher than for older men (44.8% versus 30.3% in men). The number of average years of daily tobacco use was significantly higher in men (19.8 years) than in women (14.1 years).

**TABLE 1:** Weighted characterization of study sample by gender (Santiago, Chile. 2019).

	Men		Women		*p*-value
	% or mean [95% CI]	% missing values	% or mean [95% CI]	% missing values	
Dependent variable					
Cardiovascular disease (presence) (%)	27.9 [16.2, 43.7]	0	17.7 [11.2, 26,8]	0	0.217
Independent variable					
Childhood SEP (%)					
Low SEP	37.1 [25.1, 50.8]	10.5	40.8 [33.1, 49.0]	6.8	0.720
Upper-Middle SEP	52.4 [38.1, 66.3]		52.4 [44.2, 60.5]		
Moderator variable					
ABFC (continuous) (mean)	25.8 [24.7, 27.1]	0	22.6 [21.7, 23.5]	0	<0.001
ABFC (categorical)					
Below average (%) (women ≤ 22 years; men: ≤ 25 years)	48.9 [34.9, 63.2]	0	55.0 [47.3, 62.5]	0	0.459
Above average (%) (women ≥ 23 years; men: ≥ 26 years)	51.1 [36.8, 65.1]		45.0 [37.5, 52.7]		
Covariates					
Age (years) (mean)	69.9 [69.3, 69.7]	0	69.7 [69.2, 70.3]	0	0.601
Educational level (%)					
Primary or less	30.3 [22.5, 39.3]	0	44.8 [35.4, 54.6]	0	0.041
Secondary or more	69.7 [60.7, 77.4]		55.2 [45.4, 64.6]		
Number of children	2.8 [2.5, 3.0]	0	2.8 [2.6, 3.0]	0	0.685
Arterial hypertension (presence)	69.4 [60.3, 77.1]	0	63.4 [53.4, 72.4]	0	0.332
High cholesterol (presence)	35.7 [24.3, 48.9]	0	41.9 [32.9, 51.4]	0	0.373
Daily tobacco consumption (years)	19.8 [13.0, 26.6]	0	14.1 [10.5, 17.7]	0	0.090

Note: N = 802. SEP, Socioeconomic position; ABFC, Age at birth of first child; CI, Confidence interval. *p*-value indicates the level of statistical significance of the differences between men and women (Chi square test or Wald test according to the level of measurement of the variables).

### Weighted Logistic Regression Models

Table 2 shows four weighted logistic regression models for older men (indicated by letter “a”), and four weighted models for older women (indicated by letter “b”). Models 1a and 1b include a categorical measurement of the ABFC variable. Model 1a indicates that among men, both low childhood SEP and having had their first child below the average age of their male peers is significantly associated with CVD presence (OR = 3.29, IC95% = 1.23–8.78 and OR = 2.89, IC95% = 1.08–7.74, respectively). No significant associations between these variables and CVD were observed among women (model 1b).

**TABLE 2:** Weighted logistic regression models (odds ratios) for cardiovascular disease among men and women (Santiago, Chile. 2019).

	Model 1a	Model 1b	Model 2a	Model 2b	Model 3a	Model 3b	Model 4a	Model 4b
Predictors	Men	Women	Men	Women	Men	Women	Men	Women
	OR [95% CI]	OR [95% CI]	OR [95% CI]	OR [95% CI]	OR [95% CI]	OR [95% CI]	OR [95% CI]	OR [95% CI]
Childhood SEP								
Upper-Middle (Ref.)	-	-	-	-	**-**	**-**	-	-
Low	3.29 [1.23, 8.78]*	1.10 [0.52, 2.35]	2.91 [1.05, 8.05]*	1.15 [0.51, 2.56]	10.4 [2.38, 45.46]**	0.36 [0.13, 1.01]^+^	0.04 [0.00, 0.20]**	1.98 [0.14, 28.51]
ABFC (categorical)								
Above average (Ref.)	-	-	-	-	**-**	**-**	**-**	**-**
Below average	2.89 [1.08, 7.74]*	2.05 [0.55, 7.65]	-	-	7.27 [1.47, 35.85]*	1.19 [0.40, 3.54]	**-**	**-**
ABFC (continuous)	-	-	0.95 [0.88, 1.04]	0.97 [0.85, 1.10]	**-**	-	0.86 [0.78, 0.93]***	0.98 [0.88, 1.09]
**Interaction effects**								
Childhood SEP*ABFC (categorical)								
Low*ABFC	**-**	-	**-**	-	0.11 [0.02, 0.73]*	4.89 [1.25, 19.15]*	-	-
Below average								
Childhood SEP*ABFC (continuous)								
Low*ABFC	**-**	-	**-**	-	**-**	-	1.28 [1.11, 1.48]***	0.97 [0.87, 1.09]
Constant	0.00	0.03	0.00	0.08	0.00	0.00	0.00	0.07
F Test	3.8	4.5	3.5	4.2	5.1	3.9	4.8	3.3
Pr > F	0.00	0.00	0.00	0.00	0.00	0.00	0.00	0.00
Observations	225	470	225	470	225	470	225	470

Note: OR, Odds ratios; SEP, Socioeconomic position; ABFC, Age at the birth of the first child. ABFC below average: women ≤22 years; men: ≤25 years. ABFC above average: women ≥23 years; men: ≥26 years. All models adjusted for age, educational level, number of children, arterial hypertension, high cholesterol and years of daily smoking. Weighted logistic regression models for older men are indicated by letter “a”, and weighted models for older women are indicated by letter “b”. Models 1a and 1b include childhood SEP and the categorical measurement of the ABFC. Models 2a and 2b measure the same associations as models 1a and 1b but considering the ABFC variable as a continuous measurement. Models 3a and 3b add the interaction effects between childhood SEP and ABFC (as a categorical variable) on CVD occurrence. Models 4a and 4b analyze the interaction effect between childhood SEP and ABFC (as a continuous variable) on CVD occurrence. In grey, statistically significant effects. *p* value: ***p < 0.001, **p < 0.01; *p < 0.05; + *p* < 0.10.

Models 2a and 2b measure the same associations as models 1a and 1b, but considering the ABFC variable as a continuous measurement. Among older men, low childhood SEP maintained its significant association with a higher risk of CVD (OR = 2.91, IC95% = 1.05–8.05). However, continuously measured ABFC had no statistically significant effect. Among older women, the ABFC and childhood SEP values once again showed no significant association with the risk of developing cardiovascular diseases.

Models 3a and 3b add the interaction effects between childhood SEP and ABFC (as a categorical variable) on CVD occurrence, in older women and men respectively. For men, early fatherhood mitigates the association between low SEP and CVD presence (OR = 0.11, IC 95% = 0.02–0.73). In other words, the positive association between low childhood SEP and CVD presence occurs especially among men who became fathers later than their male peers (OR = 10.40, IC 95% = 2.38–45.46). Women indicate the opposite effect: earlier motherhood among those with low childhood SEP increased CVD risk by 3.89 times (OR = 4.89, IC 95% = 1.25–19.15), while later motherhood mitigates this association (OR = 0.36, IC 95% = 0.13–1.01). However, the latter result should be considered carefully as it marginally contains in the upper part of the IC the value of the null hypothesis (that is, OR = 1.00), and consequently it is significant only at a *p* value of <0.1.

**FIGURE 2:**
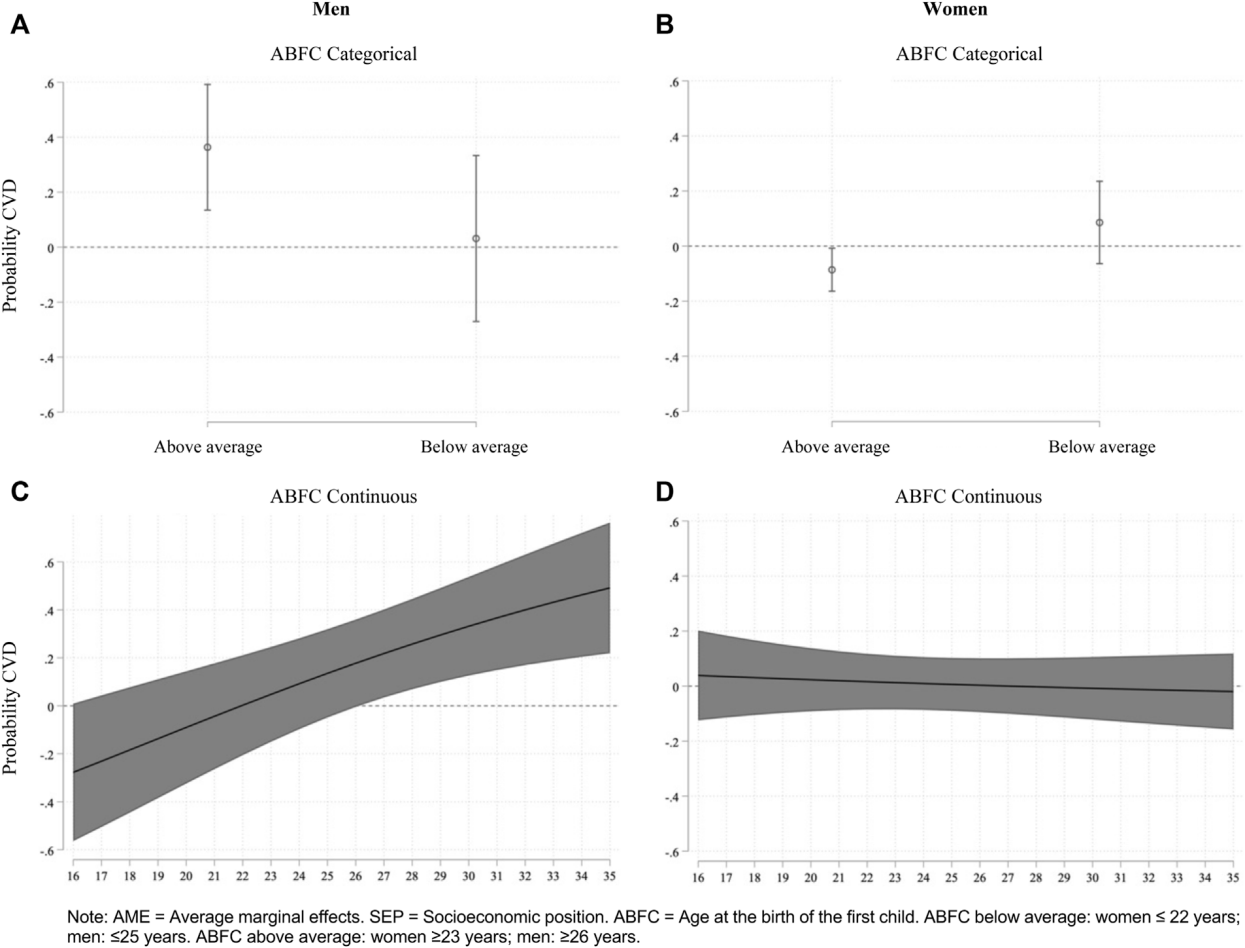
Average marginal effects of interaction effects between low childhood socioeconomic position and age at the birth of the first child (categorical and continuous) on cardiovascular diseases among men and women (Santiago, Chile. 2019). Note: AME, Average marginal effects; SEP, Socioeconomic position; ABFC, Age at the birth of the first child. ABFC below average: women ≤22 years; men: ≤25 years. ABFC above average: women ≥23 years; men: ≥26 years. **(A)** ABFC Categorical. **(B)** ABFC Categorical. **(C)** ABFC Continuous. **(D)** ABFC Continuous

As expected from these odds ratio results, when looking into the average marginal effects in Figure 2A, we notice that for men with adverse childhood socioeconomic conditions, fatherhood onset above the average age increases the CVD risks by 36.3 percentage points on average (AME = 0.363, *p* < 0.001). By contrast, Figure 2B shows that among women with low childhood SEP, motherhood onset above the average age decreases CVD risks by 8.6 percentage points on average (AME = −0.086, *p* < 0.05).

Models 4a and 4b analyze the interaction effect between childhood SEP and ABFC (as a continuous variable) on CVD occurrence, for older men and women respectively. In men, consistent with Model 3a, CVD risk associated with low childhood SEP increases significantly as fatherhood starting age increases (OR = 1.28, IC 95% = 1.11–1.48). For women, ABFC measured as a continuous variable does not significantly moderate the association between childhood SEP and CVD occurrence.

Confirming these odds ratios results, Figure 2C indicates that for men with a disadvantaged childhood socioeconomic status, whereas early fatherhood at age 16 reduces the CVD risk by 27.7 percentage points on average (AME = −0.277, *p* < 0.1), fatherhood onset at age 26 increases this risk by 17.7 percentage points (AME = 0.177, *p* < 0.1), and fatherhood onset at age 35 increases this risk by almost 50 percentage points (AME = 0.492, *p* < 0.001). On the other hand, Figure 2D shows no differences in CVD risk among women with low childhood SEP according to the age of motherhood onset (measured as a continuous variable).

## Discussion

This study shows that low childhood SEP is positively and significantly associated with cardiovascular diseases, especially among men. These findings match findings from other national and international studies [26, 27]. The greatest risk observed in men coincides with health indicators reported by Chile in recent decades, showing that men not only fall ill more often, but also die before women due to cardiovascular diseases [3, 28, 29]. Men in Chile are also less likely to attend cardiovascular health checkups in primary care centers [28]. This study therefore helps fortify the link between lower childhood SEP and worse health conditions in later life.

This study also shows that age at the birth of a first child is independently associated with CVD risk, specifically among men who became fathers before their male peers. For women, no significant results were observed. These results only partially adjust to other studies’ findings [14, 18, 19], which show an association between early parenthood and higher CVD risks for both men and women.

Finally, we provide evidence that the beginning of parenthood is a turning point for cardiovascular health in people with disadvantaged childhood SEP. By using ABFC as a categorical measurement (before and after average age for same-gender peers), we see that early motherhood significantly increased CVD risk among older women with low childhood SEP, while late motherhood decreases this risk. By contrast, older men with low childhood SEP who had their first child below their peers’ ages saw their CVD risk drop, while late fatherhood increases this risk. In a coherent way, analyzing ABFC as a continuous variable established that men with disadvantaged childhoods who delayed the birth of their first child were significantly more likely to suffer later CVD. For women, using continuously measured ABFC showed no significant results.

The fact that early fatherhood mitigates the positive association between disadvantaged childhood SEP and later CVD risk, while early motherhood amplifies this positive association, may be explained, among other reasons, by the different consequences of early parenthood within the Chilean social context which the studied cohort experienced. Specifically, the people explored in this study spent most of their lives under a “male breadwinner-female homemaker” cultural model, which is characterized by establishing a strong distinction between a male role associated with employment and public activities, and a female role focused on domestic tasks and caring for children, elderlies, partners, and other relatives [28]. In this context, one of the main consequences of the early arrival of a child (highly associated with CVD development)—namely interrupted educational paths and subsequent lower adult socioeconomic levels [16]—could have affected men less than women given the Chilean tradition that in couples with early pregnancies, priority is given to continuing the studies and careers of men [26, 30].

Furthermore, the fact that late fatherhood particularly amplifies the positive association between disadvantaged childhood SEP and CVD risk may be explained given that delaying the arrival of a first child favors the accumulation of typically masculinized cardiovascular risk behaviors (i.e. persistent alcohol consumption and tobacco use), which raise the odds of later CVD [13].

National statistical records in Chile show that over the past 4 decades, the tendency towards delaying parenthood among both women and men has consolidated, along with the persistent reduction in the average number of children [31]. Given the rise in cardiovascular risk associated with delaying fatherhood among men with low childhood SEP, it is necessary to study young cohorts in order to analyze the impact of delaying parenthood on CVD development.

### Limitations

While this research provides innovative evidence to the field of the life course determinants of later life health, there are some limitations that need to be acknowledged when interpreting its findings.

First, we point to the fact that childhood SEP was measured *via* a self-reporting measure on the economic situation of their origin family, which can limit the precision of this variable. However, additional analyses were conducted using parents’ occupation as an indicator of childhood SEP, and results were not statistically significant (results available upon request).

Second, while the data collection procedure was conducted following the latest AAPOR’s quality standards, in this study we did not consider people who died due to the CVDs explored. This might lead both to the underestimation of the actual prevalence of these diseases, and to the selection bias of socially advantaged people with a greater chance of surviving them.

Third, although we collected data using face-to-face life history calendars that were tested through cognitive interviews and usability tests, and were administered by well-trained interviewers, this corresponds to a cross-sectional quantitative questionnaire that measure both early life exposures and health status in old age in the same interview. This might lead respondents to non-random recall biases when reporting both family, health and socioeconomic statuses [32, 33].

Fourth, while the sample size of the study (*n* = 802) was large enough to represent individuals between 65 and 75 years old residing in Santiago, Chile, the number of cases in some cells when conducting interaction effects in regression models was limited, which resulted in great confidence intervals of some specific estimates.

Finally, one might argue that our analytical framework is greatly determined (or even biased) by the decision to treat the variable “age at birth of first child” as a moderator and not as a mediator variable. This is because, as current literature indicates [14], people with an economically disadvantaged childhood are more likely to become parents earlier in life than those with an economically disadvantaged position. Therefore, childhood SEP should have causal effects on ABFC. However, in Chile this is a trend especially prevalent among younger cohorts of men and women, i.e., under age 50 [34, 35]. Instead, in the cohort analyzed in this research (aged 65–75 in 2019, born between 1944 and 1954) most men and women, regardless of their socioeconomic position, transitioned to parenthood in early adulthood. Specifically, Supplementary Table S3 shows the weighted association between childhood SEP and ABFC among the whole sample, men, and women, and results are clearly ambivalent or equivocal. We observe both the absence of statistically significant differences among SEP groups in the mean ABFC among the whole sample, higher mean ABFC for low SEP among men, and higher mean ABFC for upper-middle SEP among women. However, following the recent literature in this field, we underline that these associations might be different for younger cohorts in this country.

### Conclusion

Adverse socioeconomic conditions during childhood are associated with greater development of cardiovascular diseases in later life. Parenthood onset timing is highly related with childhood SEP, as well as with the cardiovascular health of individuals. No studies have been conducted to evaluate the association between these three factors.

In this research we indicated that parenthood onset is a turning point in cardiovascular health for people with disadvantaged childhood SEP. Specifically, early motherhood increases the risk of suffering CVD among older women with a disadvantaged childhood SEP, while late motherhood decreases it. By contrast, early fatherhood decreases CVD risk among older men with an adverse childhood SEP, while late fatherhood increases it. Both the “male breadwinner-female homemaker” cultural model and masculinized health risk behaviors are plausible explanatory mechanisms for this moderator effect.

This study contributes to understanding little explored cardiovascular risk factors, which might optimize cardiovascular prevention strategies throughout life. These findings contribute to greater understanding of the role of unexplored cardiovascular risk factors such as parenthood transition timing, and therefore might be used to optimizing lifelong cardiovascular prevention strategies [36].

## Data Availability

The data used in this study are available on request from the corresponding author (i.cabib@uc.cl).
