# Supplementary material for: Childhood Socioeconomic Position and Cardiovascular Disease Among Older Women and Men: The Moderating Role of Parenthood Onset
Source: Int J Public Health. 2022 Nov 28;67:1604884. doi: 10.3389/ijph.2022.1604884 (PMC9742213; doi:10.3389/ijph.2022.1604884)
Supplement: Supplementary file 1 [file DataSheet1.PDF]

Supplementary Material

Supplementary Table S1. Weighted logistic regression models (odds ratios) for cardiovascular disease among men and women, excluding arterial hypertension, high cholesterol, and tobacco consumption (Santiago, Chile. 2019)

	Model 1	Model 2
**Predictors**	Men<br>OR<br>[95% CI]	Women<br>OR<br>[95% CI]
**Childhood SEP**		
Upper-Middle (Ref.)	-	-
Low	3.03^{+}<br>[0.92, 10.01]	1.21<br>[0.39, 3.76]
Constant	0.00	0.05
F Test	5.4	2.2
Pr > F	0.00	0.04
Observations	225	470

Note: OR = Odds ratios. SEP = Socioeconomic position. All models adjusted for age, educational level, number of children, and age at the birth of the first child. Models 1 and 2 include childhood SEP as predictor for men and women, respectively. In grey, statistically significant effects. P value: *** p <0.001, ** p <0.01; * p <0.05; + p <0.10.

Supplementary Table S2. Weighted logistic regression models (odds ratios) for cardiovascular disease among the general sample (Santiago, Chile. 2019)

	Model 1	Model 2	Model 3	Model 4
**Predictors**	OR [95% CI]	OR [95% CI]	OR [95% CI]	OR [95% CI]
**Childhood SEP**				
Upper-Middle (Ref.)	-	-	-	-
Low	1.33 [0.50, 3.52]	1.26 [0.46, 3.51]	1.29 [0.47, 3.52]	1.79 [0.48, 6.71]
**ABFC (categorical)**				
Above average (Ref.)	-	-	-	-
Below average	2.38 [0.75, 7.56]	-	-	-
**ABFC (continuous)**	-	0.97 [0.90, 1.04]	-	-
**Gender**				
Men (Ref.)	-	-	-	-
Women	-	-	0.48 [0.18, 1.25]	0.66 [0.20, 2.16]
**Interaction effects**				
**Childhood SEP*Gender**				
Low*Women	-	-	-	0.52 [0.17, 1.54]
Constant	0.00	0.00	0.00	0.00
F Test	8.0	5.9	8.8	8.4
Pr > F	0.00	0.00	0.00	0.00
Observations	697	697	743	743

Note: OR = Odds ratios. SEP = Socioeconomic position. ABFC = Age at the birth of the first child. ABFC below average: women ≤ 22 years; men: ≤25 years. ABFC above average: women ≥23 years; men: ≥26 years. All models adjusted for age, educational level, number of children, arterial hypertension, high cholesterol and years of daily smoking. Model 1 includes childhood SEP and the categorical measurement of the ABFC. Model 2 measures the same associations as model 1 but considering the ABFC variable as a continuous measurement. Model 3 adds the interaction effects between childhood SEP and ABFC (as a categorical variable) on CVD occurrence. Model 4 analyzes the interaction effect between childhood SEP and ABFC (as a continuous variable) on CVD occurrence. In grey, statistically significant effects. P value: *** p <0.001, ** p <0.01; * p <0.05; + p <0.10.

Supplementary Table S3. Weighted association between childhood socioeconomic position and age at birth of first child among the whole sample, men and women (Santiago, Chile. 2019)

	**Age at birth of first child (continuous)**					
	Whole sample		Men		Women	
**Childhood SEP**	Mean [95% CI]	p-value	Mean [95% CI]	p-value	Mean [95% CI]	p-value
Upper-Middle SEP	24.3 [23.1, 25.4]	0.361	26.2 [24.7, 27.8]	0.821	22.8 [22.2, 23.4]	0.011
Low SEP	23.5 [22.3, 24.6]		26.4 [25.1, 27.8]		21.5 [20.7, 22.2]	

Note: N = 802. SEP = Socioeconomic position. CI = Confidence interval. P-value indicates the level of statistical significance of the differences between childhood SEP and age at birth of first child, among the whole sample, among men, and among women (Wald test).
